# Preoperative first-line amlodipine in phaeochromocytoma/paraganglioma: perioperative haemodynamic instability and its predictors

**DOI:** 10.1530/EC-25-0921

**Published:** 2026-04-02

**Authors:** Chandramouli L, Saba Samad Memon, Anurag Ranjan Lila, Manjiri Karlekar, Aditya Phadte, Vijaya Sarathi, Rohit Barnabas, Sailaja Pramod Bijani, Sameer Rege, Yogesh Prabhakar Takalkar, Amala Kudalkar, Aarti Kulkarni, Nalini Shah, Tushar R Bandgar

**Affiliations:** ^1^ Department of Endocrinology and Metabolism, King Edward Memorial Hospital and Seth Gordhandas Sunderdas Medical College, Mumbai, India; ^2^ Department of Endocrinology, Vydehi Institute of Medical Sciences and Research Centre, Bangalore, India; ^3^ Intern, King Edward Memorial Hospital and Seth Gordhandas Sunderdas Medical College, Mumbai, India; ^4^ Department of General Surgery, King Edward Memorial Hospital and Seth Gordhandas Sunderdas Medical College, Mumbai, India; ^5^ Department of Anesthesiology, King Edward Memorial Hospital and Seth Gordhandas Sunderdas Medical College, Mumbai, India

**Keywords:** phaeochromocytoma, paraganglioma, amlodipine, haemodynamic instability, metanephrine phenotype, perioperative management

## Abstract

**Objectives:**

Preoperative alpha blockade is recommended for phaeochromocytomas and paragangliomas (PPGLs), whereas evidence for calcium channel blockers remains limited. This study evaluated perioperative haemodynamic outcomes with first-line amlodipine for preoperative blockade and identified predictors of intraoperative haemodynamic instability (iHDI).

**Methods/design:**

In this monocentric retrospective study, 35 operated PPGL patients who received preoperative first-line amlodipine (July 2021–March 2024) were analysed. Continuous intraoperative arterial pressure recordings were analysed for episodes and duration of hypertension, hypotension, and iHDI. Biochemical phenotype and tumour characteristics were assessed as predictors.

**Results:**

The cohort (median age: 32 years; 60% female) included 32 phaeochromocytomas and 3 sympathetic paragangliomas. Germline variants were detected in 20/34 tested patients (cluster 1: 11; cluster 2: 9). Amlodipine up to 20 mg was tolerated in all except two, with a median of 7 days to reach BP targets. Median iHDI duration was 6.67% (0–16.4). On linear regression, log-transformed plasma free metanephrine (PFMN) independently predicted iHDI duration (*β* = 2.26, *P* = 0.027). Patients with adrenergic phenotype (*n* = 13) exhibited a longer duration of SBP ≥ 160 mmHg (14 vs 1 min), a higher peak SBP (201 vs 160 mmHg), and a higher nitroglycerine dose (659 vs 59.6 μg) than those with noradrenergic phenotype. Postoperative hypotension (37.1%) was associated with a higher plasma free normetanephrine level (3,440 vs 1,242.9 ng/L) and a larger tumour size (5.1 vs 4.2 cm). No perioperative mortality occurred.

**Conclusion:**

First-line amlodipine blockade was effective in preventing iHDI in PPGLs. Plasma free metanephrine and normetanephrine correlated with intraoperative hypertension and postoperative hypotension, respectively.

## Introduction

Surgical excision is the treatment of choice for the majority of phaeochromocytomas and paragangliomas (PPGLs) (1). Intraoperative tumoural handling is typically associated with abrupt hypertensive surges, whereas profound and, sometimes, prolonged hypotension may follow tumour removal; together, these contribute to significant haemodynamic instability (HDI). With the advent of preoperative blockade and advancements in surgical and anaesthetic techniques, the mortality associated with HDI is minimised (2). The latest Endocrine Society guidelines recommend alpha blockade (phenoxybenzamine or doxazosin) followed by beta blockade as the first-line preoperative management (3), while calcium channel blockers (CCBs) are suggested as an add-on drug. A few centres have used CCBs (nifedipine or nicardipine) as the first-line drug for preoperative blockade (4). In the year 2021, a pilot, open-label, randomised controlled trial (RCT), we observed that preoperative amlodipine (*n* = 11) was associated with a significantly lower percentage time spent in intraoperative HDI (iHDI) (1.32 vs 17.8%) as compared to the gastrointestinal therapeutic system (GITS) formulation of prazosin (*n* = 9) (5). Notably, the percentage time spent in HDI with amlodipine use in this pilot RCT was also less than that observed in the only prior RCT, the PRESCRIPT trial, which reported intraoperative HDI (iHDI) of 11.1 and 12.2% with phenoxybenzamine and doxazosin, respectively (6). Since July 2021, amlodipine has been used as the first-line drug for preoperative management of secretory PPGLs at our centre. Here, we aim to analyse the perioperative haemodynamic outcomes of PPGL patients treated with amlodipine as the first-line preoperative blockade and to identify predictive factors for HDI.

## Materials and methods

This retrospective study was conducted after approval from the Institutional Ethics Committee (EC/OA-40/2024). Patients with PPGLs who had histopathological confirmation of the diagnosis, received preoperative pharmacologic blockade with first-line amlodipine, and underwent surgery at our centre between July 2021 and March 2024 were included in the study. Only those with complete perioperative haemodynamic documentation, including archived intraoperative video recordings of haemodynamic variables, were analysed. Patients with extensive metastases (*n* = 2) or who had an inferior vena cava rent during surgery (*n* = 2) were excluded because these conditions may influence HDI.

Thirty-five PPGL patients (32 phaeochromocytomas and 3 sympathetic PGLs) were included in the study, for whom baseline clinical characteristics were noted. Additionally, details of biochemical, imaging, and molecular diagnosis were recorded. Blood sample for plasma free metanephrine (PFMN) and plasma free normetanephrine (PFNMN) was drawn in a supine position (>20 min) and was assayed using liquid chromatography–tandem mass spectrometry (LC-MS/MS). The age- and sex-adjusted upper limit of normal was used to define PFNMN elevation (7). Tumours with PFMN > 61 ng/L were classified as metanephrine-secreting tumours (adrenergic phenotype, A-P); those with isolated PFNMN elevation and normal PFMN were labelled normetanephrine-secreting tumours (noradrenergic phenotype, NA-P) (7). Using the archived computed tomography (CT) scan images, tumour volume was calculated using the ellipsoid formula (π/6 × length × width × height). If a patient had multiple tumours, the sum of their volumes was used to define the final tumour volume. Germline molecular screening of genes involved in PPGLs was performed using next-generation sequencing (Illumina, USA), and data were available for 34 of the 35 patients.

The details of the preoperative amlodipine blockade, including dose, duration, and effect, were noted. Patients were blocked on either an outpatient or inpatient basis, depending on the clinician’s discretion and the patient’s preference. Amlodipine was initiated at 2.5 mg/day and gradually titrated to the maximal dose (up to 20 mg/day) if tolerated, as per the predefined protocol (Supplementary data, Fig. S1 (see section on Supplementary materials given at the end of the article)). Tolerability was defined as standing systolic BP (SBP) > 90 mmHg with adequate salt and water intake. Adequate salt intake was defined as 15 g sodium chloride per day (6). Patients were advised to increase oral fluid intake to at least 1.5 times of the daily maintenance fluid requirement (8). BP and HR were monitored at least twice daily (morning and evening) in both supine (after 10 min in supine) and upright (after 3 min of standing) positions. In patients already on other antihypertensive agents, those medications were gradually down-titrated and stopped with simultaneous up-titration of amlodipine. In select cases, if needed, additional antihypertensive drugs (telmisartan up to 80 mg/day; clonidine up to 0.3 mg/day) were added to achieve the target BP (defined as supine BP < 130/80 mmHg). Participants underwent 24-h BP monitoring (WatchBP Analyzer version 1.7.1.0), which was initiated between 24 and 48 h of reaching the target goals or the maximal tolerable dose. In patients with a resting heart rate (HR) > 90 beats/min (bpm), metoprolol up to 200 mg/day and/or ivabradine up to 15 mg/day was added to achieve a target supine HR of <90 bpm. On the day of surgery, the usual morning doses of amlodipine and/or clonidine were given, whereas telmisartan and metoprolol were withheld.

The details of the perioperative haemodynamic changes and their management were noted. Patients underwent surgery under the care of a single surgical and anaesthetic team. All patients were given 1 litre of normal saline over 12 h preceding surgery. Intra-arterial catheters were placed under local anaesthesia for continuous BP monitoring. Anaesthesia induction involved premedication with fentanyl and midazolam, followed by induction of general anaesthesia. Hypertensive episodes were primarily treated with nitroglycerine (NTG) infusion and/or boluses, with a target BP of 140/90 mmHg, and sodium nitroprusside as a secondary agent. Esmolol was used when HR exceeded 110 bpm. Hypotension was initially managed with fluid resuscitation (crystalloids to maintain CVP between 6 and 8 mmHg); if unsuccessful, noradrenaline was administered to maintain mean arterial pressure (MAP) > 60 mmHg. Postoperatively, patients were monitored and managed in the intensive care unit until they became stable, after which they were transferred to the general wards. Postoperative hypotension (PH) was defined as hypotension requiring fluid and/or administration of inotropes.

From the archived video recordings, intraoperative haemodynamic variables were noted. Primary haemodynamic variables assessed included the number of episodes and the duration of intraoperative hypertension (SBP ≥ 160 mmHg) and hypotension (MAP < 60 mmHg), as well as the duration of HDI, as defined previously (5). Additionally, other variables, such as the maximum and minimum BP and HR, the proportion of patients with various degrees of hypertension (SBP ≥ 160 mmHg, SBP ≥ 180 mmHg, and SBP ≥ 200 mmHg), hypotension (MAP < 60 mmHg), tachycardia (HR > 110 bpm), and bradycardia (HR < 50 bpm) were noted. Furthermore, the details of intraoperative vasodilators, fluids, and/or inotrope use were noted, and the haemodynamic instability (HI) score was calculated. The HI score includes measures of haemodynamic variables along with the volume of intravenous fluids and the dosage of vasoactive and vasodilator drugs administered. The HI score was adapted from the PRESCRIPT trial, and the magnesium sulphate component was replaced with NTG (9). The details of postoperative hypotension requiring fluids and/or inotropic support were noted.

### Statistical analysis

Categorical variables were expressed as *n* (%), and quantitative variables were reported as median with interquartile range (IQR) unless otherwise specified. An intergroup comparison of quantitative variables was performed using the Mann–Whitney U test for nonparametric data and the Student’s *t*-test for parametric data. Proportions were compared using the chi-square or Fisher’s exact test. Linear regression analysis was performed to identify the predictors of HDI. PFMN and PFNMN values were log-transformed prior to regression analysis to normalise their distribution and minimise skew. All statistical tests were two-sided and conducted at a significance level of *α* = 0.05. All analyses were performed using SPSS version 26.0 (IBM, USA).

## Results

The preoperative characteristics of the 35 PPGL patients (21 females), 32 of whom had phaeochromocytoma (10 with bilateral lesions) and 3 had sympathetic PGL (1 patient with two lesions), are summarised in Table 1. The median age at diagnosis was 32 (24–42) years, and six patients were younger than 20 years. Germline genetic testing was available for 34 patients, with results showing mutations in *VHL* (*n* = 7), *RET* (*n* = 7), *SDHB* (*n* = 3), *NF1* (*n* = 2), and *SDHD* (*n* = 1) genes, and was negative in 14 patients. All patients tolerated the amlodipine dose up to 20 mg, except for two, in whom postural hypotension led to a dose restriction to 5 and 10 mg. To achieve the BP target, additional antihypertensives were required in 17.1% (6 patients: telmisartan: 5; telmisartan and clonidine: 1). Drugs for HR control were required in 60% (21 patients: metoprolol: 18; metoprolol and ivabradine: 3). The median duration to reach the preoperative blockade targets was 7 (6–10) days. All patients underwent laparoscopic tumour excision except for two with 7-cm- and 9-cm-sized unilateral phaeochromocytomas, who required conversion to open surgery. The details of intraoperative haemodynamic variables are summarised in Table 2, and graphical presentations of intraoperative BP recordings for each patient are provided in the Supplementary data, Fig. S2. The median duration of surgery was 127 (96–164) minutes, with iHDI episodes (*n*) and iHDI duration (%) being 1 (0–3) and 6.67 (0–16.4), respectively. Median intraoperative NTG and noradrenaline doses were 125 (0–704.5) μg and 58.6 (0–164.6) μg, respectively, with an HI score of 54 (38–75).

**Table 1:** Preoperative characteristics of patients with PPGLs as per secretory phenotype. Data are expressed as *n* (% of total) or median (interquartile range). Statistically significant *P* values are presented in bold.

	Total cohort (*n* = 35)	Noradrenergic phenotype (*n* = 22)	Adrenergic phenotype (*n* = 13)	*P* value
Age (years)	32 (24–42)	26 (22–34)	37 (34–50)	**0.003**
Females, *n* (%)	21 (60)	12 (54.5)	9 (69.2)	0.488
Baseline haemodynamics				
Supine SBP (mmHg)	140 (126–150)	144 (125–150)	132 (126–143)	0.408
Supine DBP (mmHg)	91 (79–104)	96 (80–106)	83 (73–99)	0.106
HR (bpm)	96 (84–108)	98 (90–111)	88 (72–102)	0.053
Comorbidities, *n* (%)				
Diabetes mellitus	16 (45.7)	6 (27.2)	10 (76.9)	**0.006**
Hypertension	32 (91.4)	21 (95.5)	11 (84.6)	0.541
CAD/CVA	1 (2.8)	0	1 (7.7)	0.787
ASA risk, *n* (%)				
I	27 (77.1)	17 (77.3)	10 (76.9)	0.981
II	6 (17.1)	5 (22.7)	1 (7.7)	0.254
III	2 (5.7)	0	2 (15.3)	0.058
Biochemistry				
PFNMN (ng/L)	1,430 (793.5–3,519)	1,545 (1,020–3,559)	1,390 (577–2,380)	0.448
PFMN (ng/L)	36.2 (20.9–147.75)	23 (18.3–32.8)	157 (111–800)	<0.001
Imaging details				
Phaeochromocytoma, *n* (%)	32 (100)	19 (86.4)	13 (100)	0.163
Bilateral, *n* (%)	10 (46.6)	5 (22.7)	5 (38.5)	0.319
Sympathetic paraganglioma, *n* (%)	3 (8.5)	3 (13.6)	0	0.163
Maximum tumour size (cm)	4.8 (3.8–6.1)	4.7 (3.6–6.3)	4.9 (4.1–5.8)	0.960
Tumour volume (cm^3^)	71.05 (48.6–120.4)	86.7 (36.5–114.7)	53.81 (48.9–165.1)	0.699
Preoperative blocking details				
Maximum dose of amlodipine (20 mg) reached, *n* (%)	33 (94.2)	22 (100)	11 (84.6)	0.135
Additional drugs for BP control, *n* (%)	6 (17.1)	5 (22.7)	1 (7.7)	0.220
Duration to reach BP target (days)	7 (6–10)	8 (6–12)	7 (6–8)	0.489
Drugs for HR control, *n* (%)	21 (60)	16 (72.7)	5 (38.5)	0.075
Endpoint of blockade				
Supine SBP (mmHg)	117 (110–129)	124 (117–130)	115 (111–122)	0.081
Supine DBP (mmHg)	80 (70–84)	82 (70–85)	75 (73–80)	0.389
HR (bpm)	83 (75–92)	82 (77–90)	92 (78–101)	0.216
Standing SBP (mmHg)	117 (108–125)	119 (109–130)	110 (107–121)	0.121
Post-blockade 24 h ABPM				
Mean SBP (mmHg)	125 (114–130)	125 (114–130)	125 (119–131)	0.555
Mean DBP (mmHg)	74 (64–81)	74 (64–79)	78 (68–84)	0.139
Mean MAP (mmHg)	89 (81–93)	88 (81–92)	93 (83–97)	0.085
Mean HR (bpm)	84 (81–96)	83 (78–87)	89 (84–100)	**0.022**

ASA, American Society of Anesthesiologists; ABPM, ambulatory blood pressure monitoring; BP, blood pressure; bpm, beats per minute; CAD, coronary artery disease; CVA, cerebrovascular accident; SBP, systolic blood pressure; DBP, diastolic blood pressure; HR, heart rate; PFMN, plasma free metanephrine; PFNMN, plasma free normetanephrine; PPGLs, phaeochromocytomas and paragangliomas.

**Table 2:** Intraoperative data of patients with PPGLs as per secretory phenotype. Data are expressed as *n* (% of total) or median (interquartile range). Statistically significant *P* values are presented in bold.

	Total cohort (*n* = 35)	Noradrenergic phenotype (*n* = 22)^$^	Adrenergic phenotype (*n* = 13)*	*P* value
Surgical duration (min)	127 (96–164)	119 (98–152)	153 (85–180)	0.625
Primary haemodynamic variable				
Episodes of SBP ≥160 mmHg^a^	1 (0–3)	1 (0–2)	1 (1–3)	0.130
Duration of SBP ≥160 mmHg (min)	3 (0–15.5)	1 (0–9)	14 (1–33)	**0.045**
Episodes of MAP < 60 mmHg^b^	0 (0–1)	0 (0–0)	0 (0–1)	0.319
Duration of MAP <60 mmHg (min)	0 (0–3.5)	0 (0–0)	0 (0–5)	0.353
Episodes of HDI^c^	1 (0–3)	1 (0–3)	2 (1–3)	0.112
Duration of HDI (%)^d^	6.67 (0–16.4)	3.5 (0–9.4)	15 (2.2–24.8)	0.091
Maximum BP/HR				
Maximum SBP (mmHg)	176 (151–202)	160 (149–183)	201 (162–231)	**0.034**
Maximum MAP (mmHg)	124 (111–140)	123 (110–137)	139 (119–160)	0.113
Maximum DBP (mmHg)	106 (96.5–116.5)	106 (98–112)	110 (95–132)	0.601
Maximum HR (bpm)	105 (92–109)	106 (94–109)	102 (93–109)	0.933
Minimum BP/HR				
Minimum SBP (mmHg)	86 (78.5–100)	88 (81–99)	83 (75–100)	0.428
Minimum MAP (mmHg)	66 (59–72)	68 (60–75)	63 (56–68)	0.180
Minimum DBP (mmHg)	51 (47–57)	52 (50–59)	51 (43–53)	0.180
Minimum HR (bpm)	63 (55–71)	63 (56–72)	67 (54–70)	0.880
Number of patients *n* (%) with				
SBP ≥ 160 mmHg	21 (60)	11 (50)	10 (76.9)	0.162
SBP ≥ 180 mmHg	15 (42.8)	6 (27.3)	9 (69.2)	**0.032**
SBP ≥ 200 mmHg	5 (14.2)	1 (4.5)	4 (30.7)	**0.020**
MAP < 60 mmHg	11 (31.4)	5 (22.7)	6 (46.2)	0.258
HR > 110 bpm	8 (22.8)	5 (22.7)	3 (23.1)	1.0
HR < 50 bpm	3 (8.5)	1 (4.5)	2 (15.4)	0.541
Intraoperative antihypertensive agents				
Nitroglycerine dose (μg)^#^	125 (0–704.5)	59.6 (0–224.4)	659 (240–1,344.1)	**0.011**
Esmolol use, *n* (%)	5 (14.2)	1 (4.5)	4 (30.7)	**0.032**
Intraoperative inotropes and fluids				
Noradrenaline dose (μg)	58.6 (0–164.6)	0 (0–146)	133.2 (0–200)	0.191
Intraoperative fluids (mL)	2,000 (1,500–2,500)	2,250 (2,000–2,500)	2,000 (1,500–2,500)	0.448
Haemodynamic instability score^e^	54 (38–75)	51 (34–65)	67 (53–93)	0.121
Postoperative details				
Persistent hypotension, *n* (%)^f^	13 (37.1)	8 (36.4)	5 (38.5)	1.0
Duration of inotropes (min)	0 (0–159.5)	0 (0–120)	0 (0–192)	0.625
Fluids in the first 12 h (mL)	1,000 (500–1,500)	1,000 (500–1,500)	1,000 (500–1,500)	0.801

BP, blood pressure; bpm; beats per minute; DBP, diastolic blood pressure; HR, heart rate; HDI, haemodynamic instability; MAP, mean arterial pressure; PPGLs, phaeochromocytomas and paragangliomas; SBP, systolic blood pressure.

^a^ Each hypertensive episode was defined as a new occurrence of SBP ≥ 160 mmHg with at least 5 min of preceding SBP < 160 mmHg.

^b^ Each hypotensive episode was defined as a new occurrence of MAP < 60 mmHg with at least 5 min of preceding MAP ≥ 60 mmHg.

^c^ Episodes of HDI denote the sum of hypertensive and hypotensive episodes.

^d^ Duration of HDI % is time in SBP ≥ 160 mmHg and/or MAP < 60 mmHg as a percentage of total surgery time (from induction of anaesthesia to skin closure).

e Haemodynamic instability score was adapted from the PRESCRIPT trial, where magnesium sulphate doses were replaced with NTG dose tertiles (first tertile: 2.39 μg/kg/h; second tertile: 34.48 μg/kg/h) and noradrenaline dose tertiles (first tertile: 0.77 μg/kg/h; second tertile: 9.12 μg/kg/h).

^f^ Persistent hypotension is defined as the need for continued inotropes and/or fluids for maintaining BP postoperatively.

^#^ One patient required nitroglycerine initially and sodium nitroprusside later.

* The significance was maintained even when the adrenergic phenotype cohort was restricted to 13 patients, after exclusion of two patients in whom the maximal tolerated amlodipine dose was restricted to 5 and 10 mg, respectively.

^$^ The significance was maintained even when the noradrenergic phenotype cohort was restricted to 23 patients, after exclusion of two patients in whom the surgical approach was laparoscopic converted to open surgery.

On univariate linear regression analysis, amongst the various factors (patient, tumour, and blocking-related), maximum tumour size and log-transformed PFMN level were significant predictors of HDI duration, while on multivariate analysis, log-transformed PFMN level was the only significant predictor (*β* = 2.26, 95% CI: 0.27–4.24; *P* = 0.027) (Table 3). In comparison with patients with NA-P, those with A-P had similar baseline clinical characteristics and preoperative blockade details, except for a later age at presentation (37 vs 26 years, *P* = 0.003) and a higher prevalence of diabetes mellitus (76.9 vs 27.2%, *P* = 0.006) (Table 1). Duration of SBP ≥ 160 mmHg (14 vs 1 min), maximum SBP (201 vs 160 mmHg), and proportion of patients with SBP ≥ 180 mmHg (69.2 vs 27.3%) or 200 mmHg (30.7 vs 4.5%) were significantly higher in the A-P group (Table 2). Similarly, the median NTG dose use (659 vs 59.6 μg) and esmolol use (30.7 vs 4.5%) were significantly higher in the A-P group.

**Table 3:** Predictors of intraoperative haemodynamic instability duration (%) by univariate and multivariate analyses. Statistically significant *P* values are presented in bold.

	Univariate β (CI)	*P* value	Multivariate β (CI)	*P* value
Patient-related factors				
Age	0.19 (−0.07–0.46)	0.144	-	-
Sex (male vs female)	5.51 (−1.17–12.2)	0.103	-	-
Baseline systolic BP	0.00 (−0.14–0.14)	0.998	-	-
Baseline diastolic BP	0.05 (−0.16–0.27)	0.615	-	-
Baseline HR	0.12 (−0.06–0.3)	0.201	-	-
Presence of hypertension	0.97 (−11.21–13.16)	0.872	-	-
Presence of diabetes mellitus	4.71 (−1.93–11.36)	0.158	-	-
ASA category 1 vs 2/3	−3.42 (−4.6–11.46)	0.392	-	-
Tumour-related factors				
Maximum tumour size	1.98 (0.13–3.82)	0.036	1.92 (−0.14–3.98)	0.066
Tumour volume	−0.07 (−0.02–0.01)	0.506	-	-
Plasma free metanephrine*	2.16 (0.09–4.2)	0.041	2.26 (0.27–4.24)	**0.027**
Plasma free normetanephrine*	0.43 (−2.01–2.87)	0.719	-	-
Blocking-related factors				
Use of additional drugs for BP control	2.82 (−5.65–11.29)	0.503	-	-
Use of additional drugs for HR control	0.56 (−6.4–7.52)	0.871	-	-
Duration to reach BP target	0.42 (−0.57–1.41)	0.391	-	-

ASA, American Society of Anesthesiologists; β, regression coefficient; BP, blood pressure; CI, confidence interval; HR, heart rate.

* Values were log-transformed for regression analysis.

Thirteen of the 35 (37.1%) patients required noradrenaline and/or fluid for management of PH. On comparison of subgroups with or without PH, the former had a significantly higher PFNMN level (3,440 vs 1,242.9 ng/L) and a larger tumour size (5.1 vs 4.2 cm) (Table 4). In addition, patients with PH had a significantly lower intraoperative minimal SBP (78 vs 90 mmHg), minimal DBP (45 vs 53 mmHg), and HR (57 vs 66 bpm), as well as required higher intraoperative fluids (2,500 vs 2,000 mL) and an noradrenaline dose (153.4 vs 0 μg) with a higher HI score (67 vs 51) (Supplementary data, Table S3). Post-surgical complications, such as surgical site infection, atelectasis, and fever, occurred in one patient each. There was no perioperative mortality.

**Table 4:** Baseline characteristics of patients with PPGLs with and without postoperative hypotension. Data are represented as *n* (% of total) or median (interquartile range). Statistically significant *P* values are presented in bold.

	Postoperative hypotension (*n* = 13)	No postoperative hypotension (*n* = 22)	*P* value
Age (years)	34 (24–37)	32 (24–45)	0.601
Females, *n* (%)	7 (50)	14 (61.5)	0.724
Baseline haemodynamics			
SBP (mmHg)	127 (117–147)	144 (134–152)	0.130
DBP (mmHg)	95 (81–106)	90 (78–101)	0.489
HR (bpm)	100 (90–108)	96 (82–107)	0.271
Comorbidities, *n* (%)			
Diabetes mellitus	6 (50)	10 (46.1)	1.000
Hypertension	11 (85.7)	21 (96.1)	0.541
CAD/CVA	1 (7.14)	0	0.350
ASA risk, *n* (%)			
I	10 (76.9)	17 (77.3)	0.981
II	1 (7.7)	5 (22.7)	0.254
III	2 (15.4)	0	0.058
Biochemistry			
PFNMN (ng/L)	3,440 (1,820–4,520)	1,242.9 (571.8–1,907.5)	**0.004**
PFMN (ng/L)	39 (28.8–552)	25 (18.7–104.9)	0.123
Imaging details			
Phaeochromocytoma, *n* (%)	12 (92.8)	20 (92.3)	1.000
Bilateral, *n* (%)	4 (28.6)	6 (30.8)	1.000
Sympathetic paraganglioma, *n* (%)	1 (7.14)	2 (7.69)	0.278
Maximum tumour size (cm)	5.1 (4.8–7)	4.2 (3.23–5.5)	**0.012**
Tumour volume (cm^3^)	111.5 (49.8–134.2)	58.5 (26.7–105.2)	0.216
Preoperative blocking details			
Maximum dose of amlodipine (20 mg) reached, *n* (%)	12 (92.8)	21 (100)	1.000
Additional drugs for BP control, *n* (%)	3 (28.6)	4 (30.8%)	1.000
Duration to reach BP target (days)	7 (6–10)	7 (6–9)	0.880
Drugs for HR control, *n* (%)	9 (71.4)	12 (53.8)	0.488
Endpoint of blockade			
Supine SBP (mmHg)	118 (114–130)	121 (116–127)	0.724
Supine DBP (mmHg)	80 (70–84)	80 (71–84)	0.699
HR (bpm)	82 (72–91)	87 (78–97)	0.749
Standing SBP (mmHg)	118 (107–125)	117 (110–125)	0.353

ASA, American Society of Anesthesiologists; BP, blood pressure; bpm, beats per minute; CAD, coronary artery disease; CVA, cerebrovascular accident; SBP, systolic blood pressure; DBP, diastolic blood pressure; HR, heart rate; PFMN, plasma free metanephrine; PFNMN, plasma free normetanephrine; PPGLs, phaeochromocytomas and paragangliomas.

## Discussion

In this monocentric experience, including 35 PPGL patients who received first-line amlodipine blockade preoperatively, iHDI episodes were infrequent (median episodes: 1) and brief (median: 6.67% of operative time), and PFMN level was an independent predictor of iHDI duration. PH was seen in about one-third of patients, and those with PH had a significantly larger tumour size and higher PFNMN levels.

CCBs blunt catecholamine-induced vasoconstriction by inhibiting noradrenaline-mediated calcium influx into vascular smooth muscle, acting distally to the α-adrenergic receptor at the final common pathway of vasoconstriction (10). In a French study, preoperative nifedipine followed by intraoperative nicardipine provided reasonable control of systemic vascular resistance throughout the PPGL surgery (4). Few studies have evaluated the use of CCBs as preoperative blockade in PPGL patients (4, 10, 11). In a pilot, open-label RCT conducted at our centre, preoperative amlodipine (*n* = 11) was associated with a significantly shorter percentage time in HDI (1.32%) than prazosin GITS (*n* = 9, 17.8%), and the study concluded that further data are needed to substantiate these findings (5). Here, we report our centre’s experience with preoperative amlodipine blockade in 35 patients with secretory PPGLs (13 A-P), the majority undergoing laparoscopic surgery, achieving a median 6.67% time in iHDI. This is slightly higher than that noted in the amlodipine arm of the previous RCT (1.32%). However, this iHDI duration is shorter than that reported in the PRESCRIPT trial (phenoxybenzamine: 11.1% and doxazosin: 12.2%) (6). 17.1% of patients required additional antihypertensives in our study as compared to 42.4 and 39.7% in phenoxybenzamine and doxazosin arms of the PRESCRIPT trial (6). Ivabradine, used in a small subset of patients in addition to metoprolol, reduces heart rate without negative inotropy and may be a useful adjunct in selected cases (12). An additional advantage of our regimen was lower drug-related orthostasis (20 vs ∼40%), allowing faster drug up-titration and, consequently, a shorter median duration of blockade (7 vs ∼14 days) compared with the PRESCRIPT trial (6). Thus, our data strengthen the evidence supporting the efficacy of amlodipine for preventing iHDI in PPGLs.

Various study cohorts using preoperative alpha blockade have reported that tumour size and plasma/urine catecholamines and their metabolites are associated with iHDI (13, 14, 15, 16, 17, 18, 19, 20). In our study, PFMN level was the only predictor of iHDI on multivariate analysis, and PPGL patients with A-P had a significantly higher intraoperative maximal SBP (201 vs 160) than those with NA-P. One study from Japan using preoperative alpha blockade reported that urinary epinephrine and MN levels were associated with iHDI, and two studies from China using preoperative alpha blockade reported an association between iHDI and cluster 2 mutations (13, 14, 15). In our study, the difference in iHDI between A-P and NA-P was mainly due to the duration of intraoperative hypertension despite a higher NTG dose in the A-P subgroup. In a French study using nicardipine, sustained BP elevations during tumour handling were present despite controlled systemic vascular resistance; these were associated with a high cardiac index (10). This phenomenon is likely to be more pronounced in tumours with A-P, as tumour-handling-related epinephrine release preferentially stimulates cardiac β_1_-adrenergic receptors, producing inotropic and chronotropic effects that increase cardiac output and produce hypertensive surges that persist despite blunting of α1-mediated vasoconstriction achieved with CCBs or alpha blockers. As a result, the cardiac-driven component of hypertension remains insufficiently controlled, explaining the more frequent and severe iHDI observed in epinephrine-producing PPGLs. Hence, from a clinical perspective, it should be noted that intraoperative hypertension was effectively controlled with CCBs in both noradrenergic and adrenergic phenotypes, although control appeared more favourable in the noradrenergic subgroup. Further studies are needed to confirm our findings, and if proven so, potential strategies that can further reduce intraoperative hypertension in patients with adrenergic phenotypes are warranted.

Few study cohorts using preoperative alpha blockade have reported the prevalence of PH ranging from 28 to 46% and the association of PH with tumour size and higher baseline urine epinephrine and norepinephrine (21, 22). In our cohort using preoperative amlodipine blockade, 37.1% had PH, which is comparable to the PRESCRIPT trial (38.8–40%). Patients with PH had larger tumours and higher preoperative PFNMN levels (not PFMN levels). The PH group also exhibited lower intraoperative nadir values for SBP, DBP, and HR. Multivariate logistic regression for PH predictors was not feasible due to the modest sample size and low event rate. These preliminary observations suggest that a greater basal noradrenergic drive may be associated with potential postoperative vasoplegia, possibly due to downregulation of peripheral vascular noradrenergic receptors. Hence, from a clinical perspective, this suggests the importance of anticipating vasoplegia, particularly in patients with large noradrenergic tumours, and further studies are required to confirm these observations.

Data on the use of amlodipine in PPGLs remain limited and are largely confined to our prior RCT. Here, we report real-world data on an additional 35 patients with PPGLs who received first-line amlodipine. The strength of this study is the use of a uniform preoperative blockade, surgical and anaesthetic care regimen, and continuous intra-arterial BP/HR recording for objective HDI assessment. Limitations include its retrospective design, modest sample size, and lack of a comparative arm with α-blockers. Moreover, the HI score, adapted from PRESCRIPT with substitution of magnesium sulphate by nitroglycerine, is not formally validated and should be interpreted cautiously. Nevertheless, to the best of our knowledge, our data constitute the largest PPGL cohort with first-line amlodipine blockade, reporting iHDI and PH rates, along with their predictors.

## Conclusion

In this monocentric study, first-line amlodipine blockade was effective in preventing iHDI in PPGLs. Plasma free metanephrine and normetanephrine levels correlated with intraoperative hypertension and postoperative hypotension, respectively. This study supports the use of amlodipine as an effective alternative first-line drug for the preoperative management of secretory PPGLs. However, further large, multicentric RCTs are warranted in this regard.

## Supplementary materials



## Declaration of interest

There are no conflicts of interest related to this study. The authors have nothing to disclose.

## Funding

The authors received no financial support for the research, authorship, and/or publication of this article.

## Data availability

The data that support the findings of this study are available on request from the corresponding author. The data are not publicly available due to privacy or ethical restrictions.

## Statement of ethics

This study was performed in accordance with the Declaration of Helsinki and approved by the Institutional Ethics Committee of our centre (reference number EC/OA-40/2024). Because this was a retrospective study, obtaining informed consent from participants was not required, and the Institutional Ethics Committee granted a waiver for it.
