# Supplementary material for: Preoperative first-line amlodipine in phaeochromocytoma/paraganglioma: perioperative haemodynamic instability and its predictors
Source: Endocr Connect. 2026 Apr 2;15(4):e250921. doi: 10.1530/EC-25-0921 (PMC13052797; doi:10.1530/EC-25-0921)
Supplement: Supplementary file 1 [file supplementary_materials.pdf]

Supplementary data, figure S1: Preoperative blocking protocol

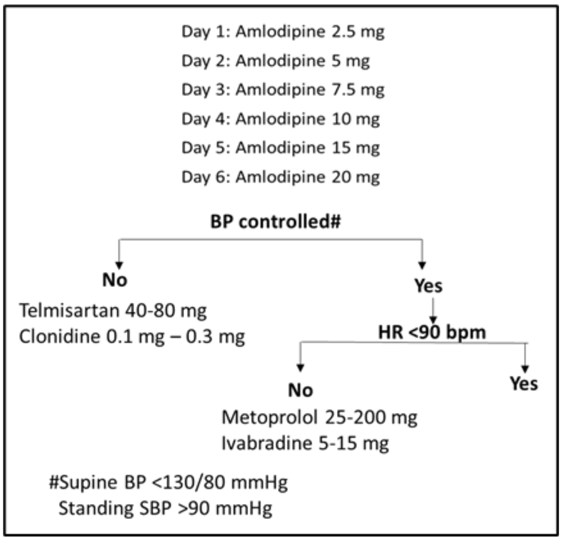

Abbreviations: BP, blood pressure; bpm, beats per minute; HR, heart rate.
#Supine BP <130/80 mmHg and Standing SBP >90 mmHg

Supplementary data, figure S2: Intraoperative blood pressure recordings for individual patients. Panels 1–22 represent patients with a noradrenergic phenotype, and Panels 23–35 represent patients with an adrenergic phenotype.

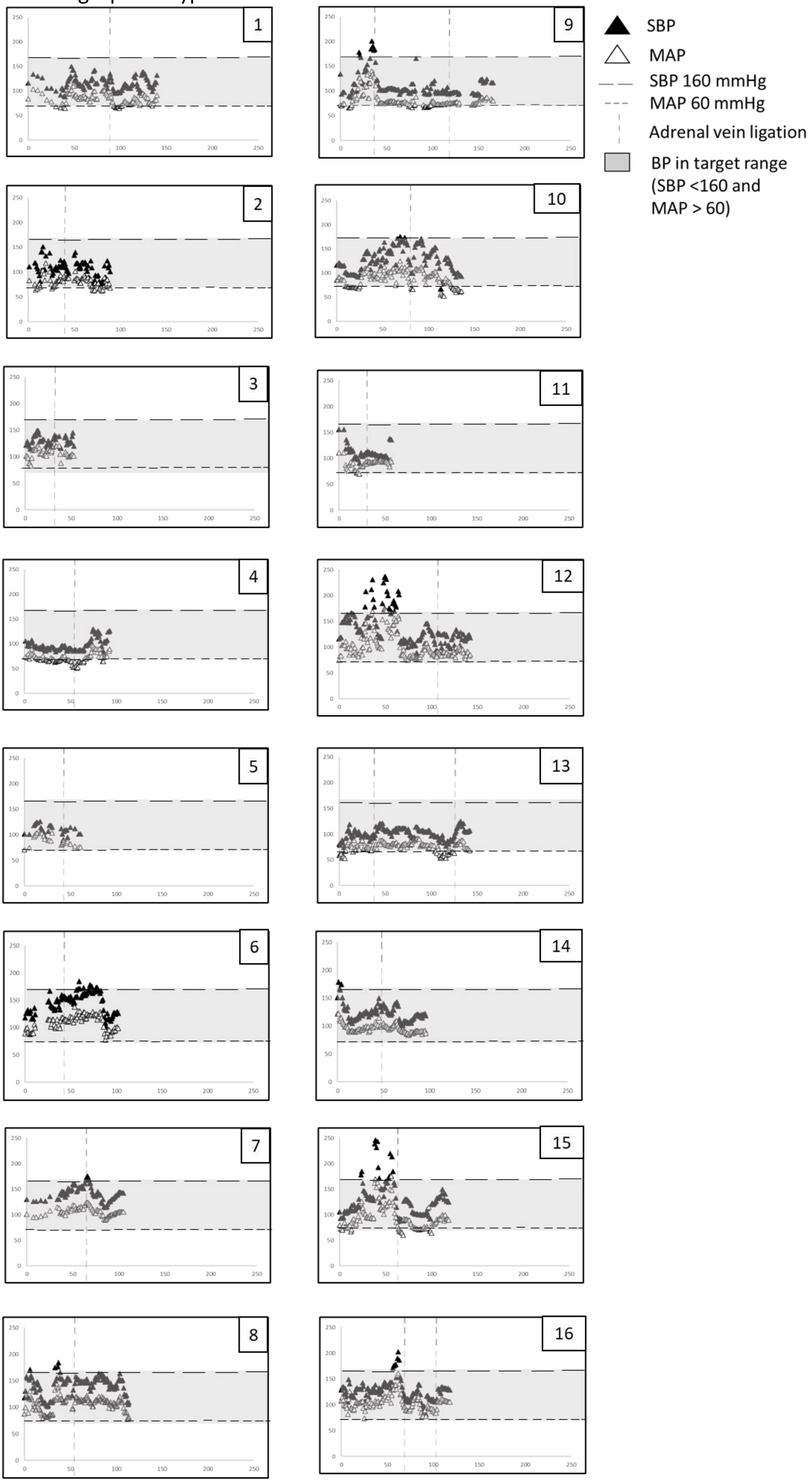

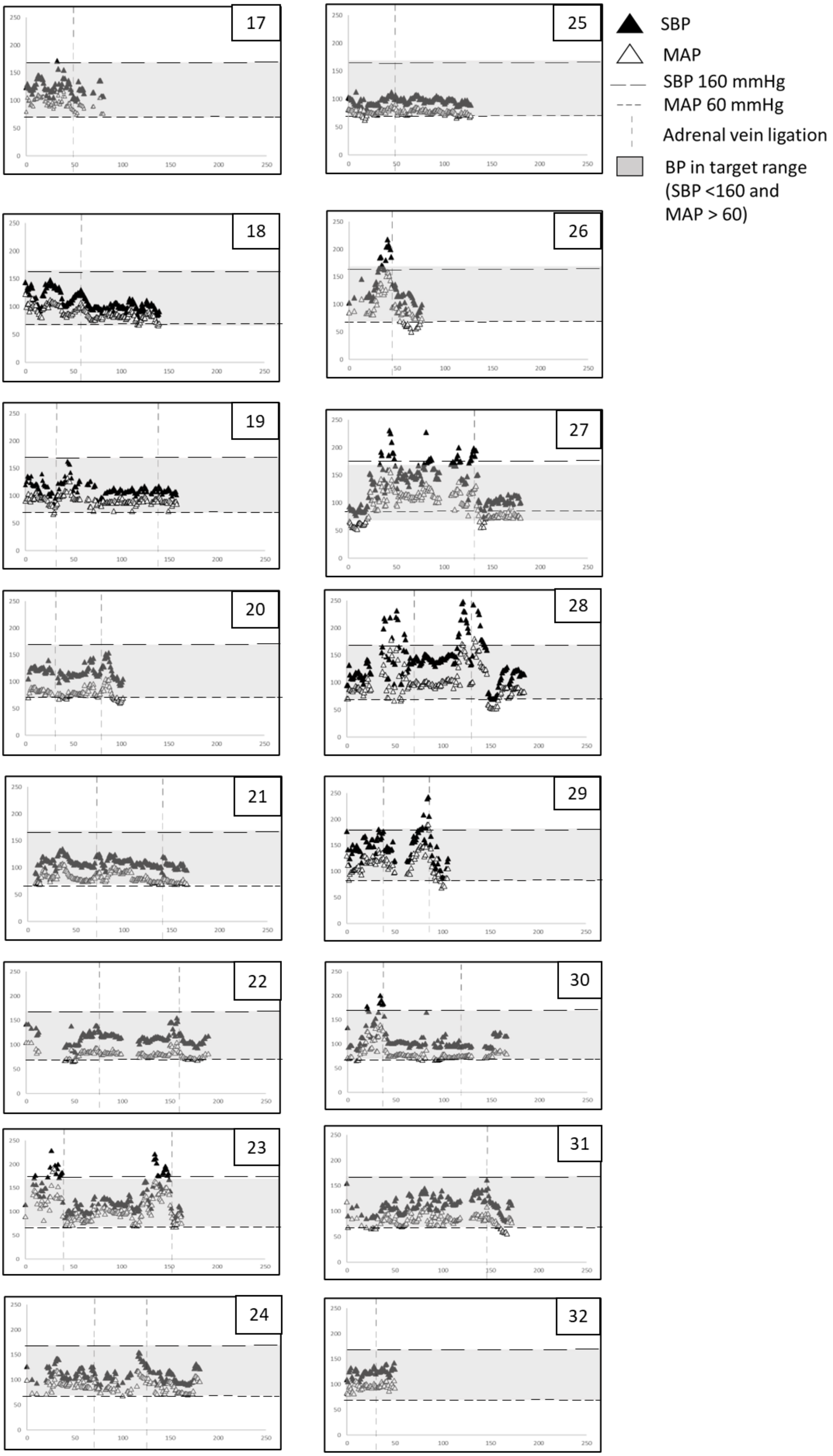
17
25
18
26
19
27
20
28
21
29
22
30
23
31
24
32
SBP
MAP
SBP 160 mmHg
MAP 60 mmHg
Adrenal vein ligation
BP in target range
(SBP <160 and
MAP > 60)

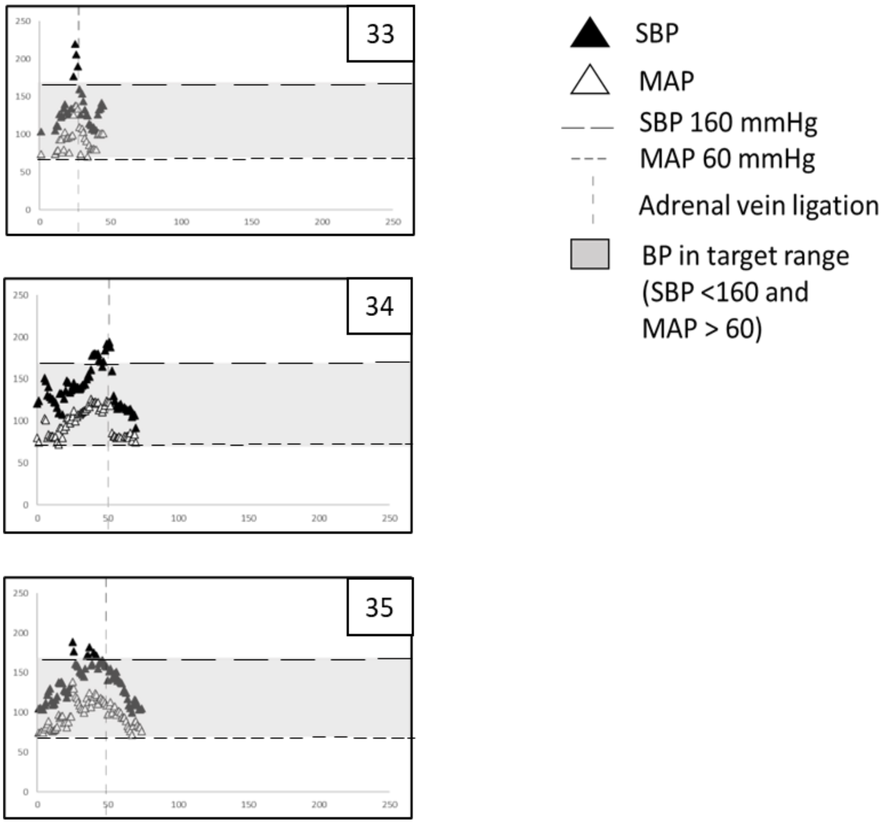
33
34
35
250
200
150
100
50
0
0
50
100
150
200
250
SBP
MAP
SBP 160 mmHg
MAP 60 mmHg
Adrenal vein ligation
BP in target range
(SBP <160 and
MAP > 60)

Supplementary data, table S3: Perioperative hemodynamics of patients with PPGL with and without postoperative hypotension

	**Patients with PH (n=13)**	**Patients without PH (n=22)**	**p value**
Surgical duration (min)	155 (127-180)	109 (86-140)	0.625
Primary hemodynamic variable			
Episodes of SBP ≥160 mmHg [a]	1 (0-3)	1 (0-2)	0.203
Duration of SBP ≥160 mmHg (min)	7 (0-33)	3 (0-11)	0.169
Episodes of MAP < 60 mmHg [b]	0 (0-1)	0 (0-0)	0.243
Duration of MAP <60 mmHg (min)	0 (0-5)	0 (0-0)	0.203
Episodes of HDI [c]	2 (1-4)	1 (0-2)	0.478
Duration of HDI (%) [d]	9.16 (0.9-20.47)	5.23 (0-13.2)	0.271
Maximum BP/HR			
Maximum SBP (mmHg)	176 (151-231)	170 (150-195)	0.448
Maximum MAP (mmHg)	125 (116-161)	124 (109-139)	0.448
Maximum DBP (mmHg)	105 (100-132)	107 (95-112)	0.319
Maximum HR (bpm)	102 (84-115)	106 (101-109)	0.827
Minimum BP/HR			
Minimum SBP (mmHg)	78 (75-83)	90 (85-102)	**0.003**
Minimum MAP (mmHg)	62 (53-70)	67 (60-72)	0.229
Minimum DBP (mmHg)	45 (40-51)	53 (51-58)	**0.007**
Minimum HR (bpm)	57 (52-66)	66 (60-72)	**0.045**
Number of patients n (%) with			
SBP ≥ 160 mmHg	9 (71.4%)	12 (57.7%)	0.488
SBP ≥ 180 mmHg	6 (50%)	9 (42.3%)	1.000
SBP ≥ 200 mmHg	5 (42.8%)	5 (26.9%)	0.444
MAP < 60 mmHg	6 (50%)	5 (26.9%)	0.258
HR > 110 bpm	5 (35.7%)	3 (19.2%)	0.116
HR < 50 bpm	3 (21.4%)	0	**0.044**
Intraoperative antihypertensive agents			
Nitroglycerin dose (µg) [#]	300 (42-891.6)	104.16 (0-252.51)	0.216
Esmolol use, n (%)	1 (7.7%)	4 (18.2%)	0.734
Intraoperative inotropes and fluids			
Noradrenaline dose (µg)	153.4 (119.97-192)	0 (0-64.5)	**0.010**
Intraoperative fluids (ml)	2500 (2000-2500)	2000 (1500-2500)	**0.034**
Hemodynamic instability score [e]	67 (54-84)	51 (34-64)	**0.045**
Postoperative details			
Duration of inotropes (min)	280 (130-1000)	0 (0-0)	**<0.001**
Fluids in the first 12 hours (ml)	2000 (1500-4000)	500 (500-1000)	**<0.001**

Data expressed as n (% of total) or median (interquartile range).

Abbreviations: BP, blood pressure; bpm; beats per minute; DBP, diastolic blood pressure; HR, heart rate; HDI, hemodynamic instability; MAP, mean arterial pressure; PH, postoperative hypotension; PPGL, pheochromocytomas and paragangliomas; SBP, systolic blood pressure.

[a]Each hypertensive episode was defined as a new occurrence of SBP ≥ 160 mmHg with at least 5 minutes of preceding SBP < 160 mmHg.
[b]Each hypotensive episode was defined as a new occurrence of MAP < 60 mmHg with at least 5 minutes of preceding MAP ≥ 60 mmHg.
[c]Episodes of HDI denotes the sum of hypertensive and hypotensive episodes.
[d]Duration of HDI is time in SBP ≥ 160 mmHg and/or MAP < 60 mmHg as a percentage of total surgery time (from induction of anesthesia to skin closure).
[e]Hemodynamic instability score was adapted from PRESCRIPT trial where magnesium sulfate doses were replaced with NTG dose tertiles (first tertile: 2.39 μg/kg/hr, second tertile: 34.48 μg/kg/hr) and Noradrenaline dose tertiles (first tertile: 0.77 μg/kg/hr, second tertile: 9.12 μg/kg/hr)
[#] 1 patient required nitroglycerin initially and sodium nitroprusside later
